# Illuminating microRNA Transcription from the Epigenome

**DOI:** 10.2174/138920213804999183

**Published:** 2013-03

**Authors:** Praveen Sethupathy

**Affiliations:** 5091 Genetic Medicine Building, 120 Mason Farm Road, Department of Genetics, Lineberger Comprehensive Cancer Center, Carolina Center for Genome Sciences, The University of North Carolina at Chapel Hill, Chapel Hill, NC 27599, USA

**Keywords:** Chromatin, complex disease, epigenome, genomics, microRNA, nascent RNA, promoter, transcription.

## Abstract

Cellular gene expression is governed by a complex, multi-faceted network of regulatory interactions. In the last decade, microRNAs (miRNAs) have emerged as critical components of this network. miRNAs are small, non-coding RNA molecules that serve as post-transcriptional regulators of gene expression. Although there has been substantive progress in our understanding of miRNA-mediated gene regulation, the mechanisms that control the expression of the miRNAs themselves are less well understood. Identifying the factors that control miRNA expression will be critical for further characterizing miRNA function in normal physiology and pathobiology. We describe recent progress in the efforts to map genomic regions that control miRNA transcription (such as promoters). In particular, we highlight the utility of large-scale “-omic” data, such as those made available by the ENCODE and the NIH Roadmap Epigenomics consortiums, for the discovery of transcriptional control elements that govern miRNA expression. Finally, we discuss how integrative analysis of complementary genetic datasets, such as the NHGRI Genome Wide Association Studies Catalog, can predict novel roles for transcriptional mis-regulation of miRNAs in complex disease etiology.

## INTRODUCTION

MicroRNAs (miRNAs) are short (~22 nt) non-coding RNAs that regulate gene expression at the post-transcriptional level. They have been identified as: (i) stable plasma biomarkers for various disorders [1], (ii) factors that underlie genetic predisposition toward several diseases (Sidebar 1), and (iii) promising targets of novel therapeutic strategies [2, 3]. Extensive computational and experimental research in the last decade has substantially improved our understanding of the mechanisms underlying miRNA-mediated gene regulation [4-7]. However, transcriptional control of the miRNAs themselves is poorly characterized, and merits further investigation [8-11].

**Fig. (1):**
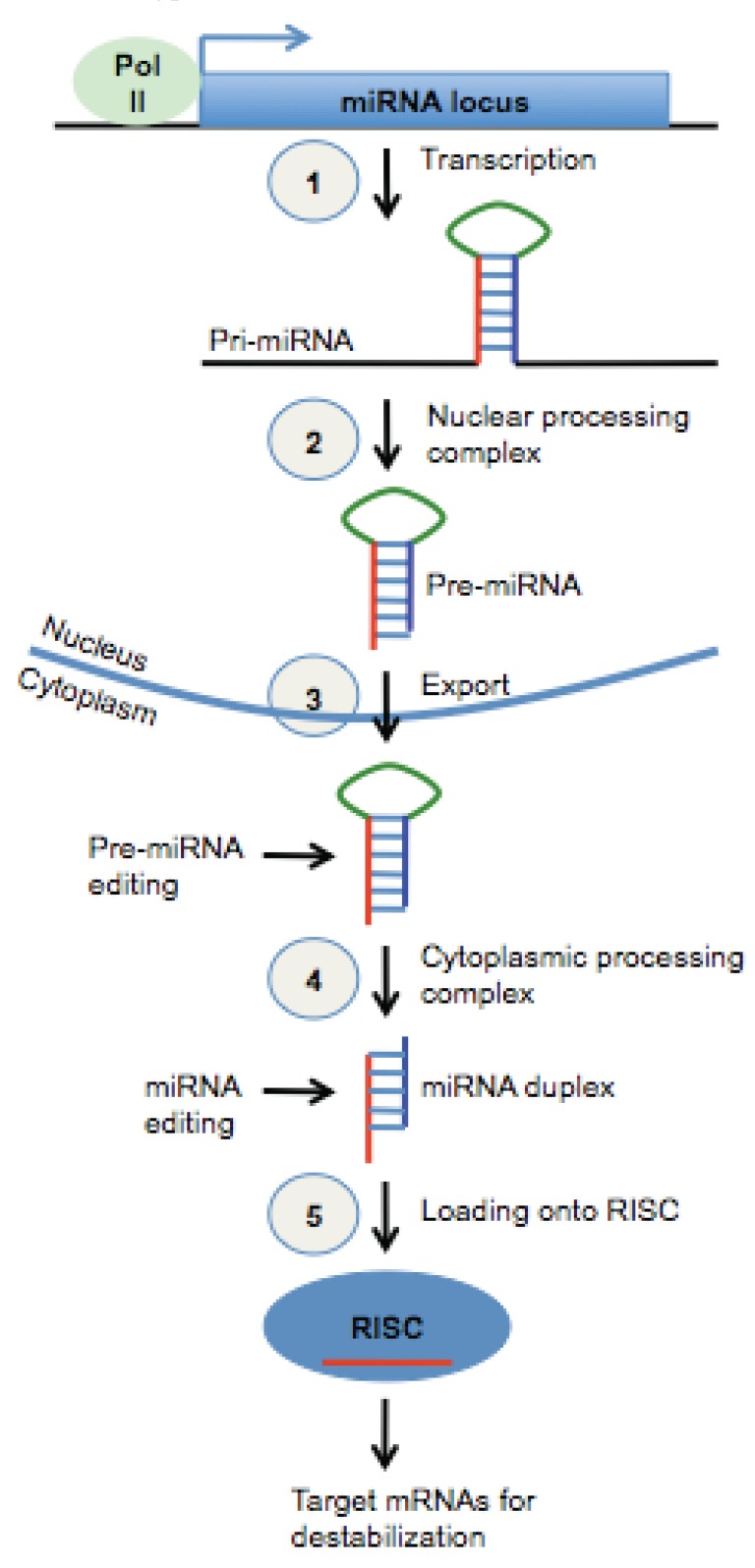
**The canonical miRNA biogenesis pathway.** (**1**) RNA polymerase II/III transcribes a primary miRNA transcript (pri-miRNA) which contains one or more hairpin-like structures (pre-miRNA); (**2**) The pri-miRNA is processed by a nuclear protein complex, liberating a pre-miRNA; (**3**) The pre-miRNA is exported to the cytoplasm; (**4**) The pre-miRNA is further processed by a cytoplasmic protein complex yielding a ~22 nt miRNA duplex; (**5**) One strand of the duplex is loaded onto the RNA Induced Silencing Complex (RISC), which it guides to target mRNAs for mRNA degradation and/or translational repression.

miRNAs are processed from primary transcripts (pri-miRNAs) that are predominantly transcribed by RNA Polymerase II (Fig. **1**). The pri-miRNA harbors one or more regions where the underlying sequence folds into a hairpin-like loop structure, which is referred to as the precursor miRNA (pre-miRNA). The pre-miRNA is processed by the nuclear Microprocessor Complex, which includes the RNase III enzyme Drosha, and exported to the cytoplasm in an energy-dependent manner. In the cytoplasm, the pre-miRNA is cleaved by the endoribonuclease Dicer and its protein partners to yield a ~22bp RNA duplex. Dicer also facilitates the incorporation of one of the strands of this duplex, which is the mature miRNA, into the RNA Induced Silencing Complex (RISC). Stable base pairing between a miRNA and a target messenger RNA (mRNA) mediates the tethering of the RISC to the mRNA. The RISC then employs one or more of several strategies to inhibit mRNA translation [5, 12].

The details of this canonical biogenesis pathway (Fig. **1**), from pri-miRNA to miRNA-RISC, were obtained through extensive interdisciplinary biological research during the last decade [13]. However, pri-miRNA transcription, which sets the entire biogenesis pathway into motion, remained largely uncharacterized in large part due to the difficulty of mapping miRNA transcription start sites and the immediately up-stream promoter regions. As is the case for any mRNA transcribed from a protein-coding gene, the 5’-end of a pri-miRNA corresponds to the active transcription start site (TSS). However, due to the rapid rate of pri-miRNA turnover (processing and degradation), conventional methods for detecting the 5’-end of a transcript (such as cDNA cloning or 5’-rapid amplification of cDNA ends) are often unproductive [14]. Reducing the efficacy of nuclear pri-miRNA processing by knockdown/knockout of the relevant enzymes (such as Drosha), thereby increasing the stability of the pri-miRNA, has helped in some instances [14-17]. However, this approach has not been reliable because it induces dramatic global down-regulation of mature miRNAs, which can lead to diminished cell viability.

Several helpful computational strategies for pri-miRNA characterization have been developed in the last few years [18-24]. Three of the most recent efforts, from Saini et al. [22]; Wang et al. [23]; and Wang et al. [24], involved comprehensive genome-wide scans for known and predicted features of transcription start [22-24] and end regions [22]. These in silico analyses offered new predictive insights into miRNA promoters and pri-miRNA structure, which provided further impetus for large-scale experimental validation in diverse cell types.

**Fig. (2):**
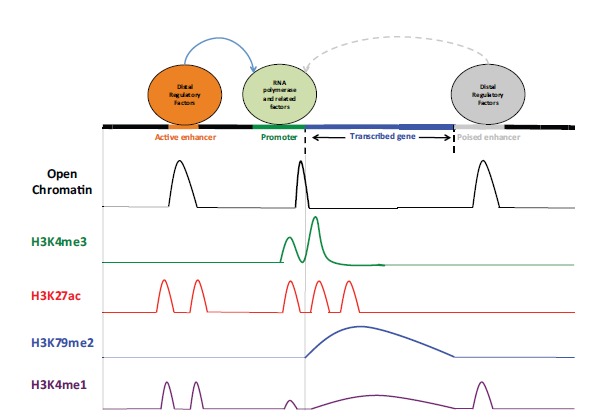
**Epigenomic profiling.** Specific combinations of chromatin marks denote different classes of functional elements, including poised enhancers, active enhancers, transcribed loci, and promoters. For example, promoters are enriched for H3K4me3, H3K27ac, and H3K79me2; active enhancers are depleted for H3K4me3 and enriched for H3K27ac.

Recent experimental efforts to map miRNA promoters, and full-length pri-miRNAs, circumvent the need to access the pri-miRNAs themselves, by analysis of specific epigenomic/chromatin markers [25]. The basic unit of chromatin is the nucleosome, which consists of ~150bp of DNA wrapped around an octamer of specialized proteins called histones. Histones are subject to a vast array of chemical modifications. Combinations of different histone modifications denote different classes of functional elements, including promoters (Fig. **2**). These chromatin marks are not directly influenced by pri-miRNA turnover dynamics; as such, they provide the basis for a compelling strategy for the discovery of miRNA promoters.

## EPIGENOMIC DATA ANALYSIS FOR THE DISCOVERY OF miRNA PROMOTERS

**Fig. (3):**
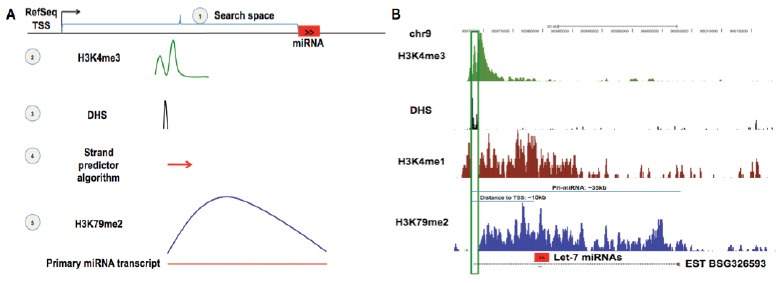
(**A**)** miRNA promoter identification using epigenomic profiles.** (**1**) Define the search space as the genomic region between the 5’ end of a mature miRNA and the nearest RefSeq annotated transcription start site (TSS), (**2**) Scan for H3K4me3 peaks, (**3**) If one or more are identified, assess each for the presence of overlapping DHS peaks, (**4**) Apply the “strand predictor” algorithm to assign an orientation to the H3K4me3 peak, (**5**) If this predicted orientation matches that of the mature miRNA, assess whether the H3K79me2 signal extends from the H3K4me3 peak to at least the end of the mature miRNA. If so, then the DHS peak will be designated as the candidate TSS for the mature miRNA in consideration. (**B**) **Candidate islet-active TSS for the primary transcript of the widely-expressed let-7a-1/7d/7f-1 miRNA cluster.** The TSS (green box; DHS+, H3K4me3+, H3K4me1-) is ~10kb upstream of the 5’-most microRNA in the cluster (red box), and the full-length primary transcript (H3K79me2+) of ~35kb matches a known expressed sequence tag (EST BSG326593). This EST likely represents a non-coding RNA transcript from which the let-7 miRNAs are processed.

Several recent studies [17, 26-30], including one of our own [30], have identified miRNA promoters in different cell types by performing genome-wide profiling of promoter-associated chromatin marks via DNase I hypersensitivity (DHS) mapping [31] and chromatin immunoprecipitation (ChIP) followed by large-scale microarray analysis (DHS/ChIP-chip) or next-generation sequencing (DHS/ChIP-seq)[32]. DHS mapping identifies sites of open chromatin that are accessible to factors that influence gene expression. Active promoters are characterized by open chromatin regions enriched for both the histone H3 lysine 4 trimethylation (H3K4me3 peaks) and the histone H3 lysine 79 di-methylation marks (Fig. **2**). We recently performed DHS-seq and ChIP-seq to profile open chromatin, H3K4me3 and H3K79me2 across the epigenome of the human pancreatic islet [30]. Using these data, which can be downloaded from the Gene Expression Omnibus (GEO) repository (http://www.ncbi.nlm.nih.gov/geo/) with the accession number GSE23784, we identified > 10,000 active promoter regions in the human islet [30]. To pinpoint promoters of miRNAs from among these, we implemented the following five-step strategy (Fig. **3A**):

1. Define the search space as the genomic region between the 5’-end of a mature miRNA and the nearest up-stream RefSeq-annotated transcription start site (TSS);
2. Scan the space for H3K4me3 peaks;
3. If one or more are identified, assess each for the presence of overlapping regions of open chromatin (DHS peaks);
4. Predict promoter orientation based on relative positioning of the DHS and H3K4me3 peaks. DHS peaks tend to be punctate around the TSS, while H3K4me3 peaks are broader and extend well into the body of the transcription unit (Fig. **3A**). In a previous analysis of known promoters of islet-expressed protein-coding genes [30], we found that the location of the DHS peak relative to the H3K4me3 peak predicts the orientation/directionality of the underlying promoter with ~90% accuracy;
5. Finally, if the predicted orientation matches that of the mature miRNA, assess whether the signal for histone H3 lysine 79 dimethylation (H3K79me2), which denotes actively transcribed regions, extends from the H3K4me3 peak to at least the 3’-end of the mature miRNA.

**Fig. (4):**
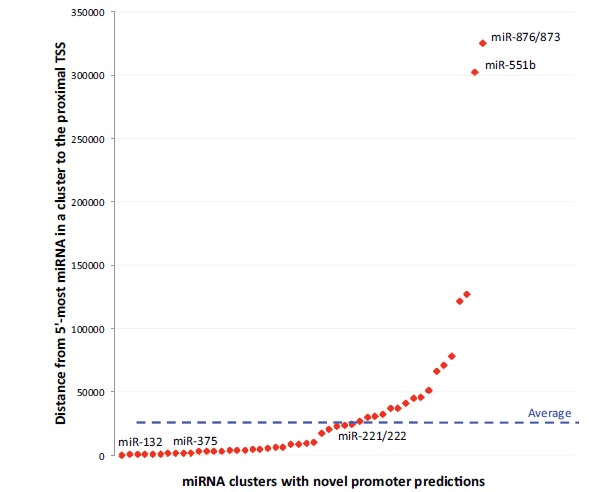
**miRNA promoters in human pancreatic islets.** Promoter regions were identified for 50 pri-miRNAs (red data points). Y-axis depicts the distance (base pairs; bp) between the identified promoter and the 5’-most mature miRNA in the pri-miRNA. Blue dashed line depicts the average distance, ~35,000 bp. Strikingly, two miRNAs, miR-876/873 and miR-551b, are located more than 300,000 bp downstream of their promoters.

This approach identified novel promoter regions (i.e. not previously annotated and not shared with a protein-coding gene) for ~50 pri-miRNAs in human pancreatic islets (Table **1**; Fig. **4**). These included: (1) 37 that overlapped computationally predicted transcription start sites according to Eponine [33]; (2) 16 that corresponded to start sites of annotated expressed sequence tags (ESTs) (e.g. Fig. **3B**); and (3) 5 of 8 previously mapped pri-miRNA promoters, such as the one for pri-miR-21[34]. The average distance between an islet pri-miRNA promoter and the nearest (5’-most) mature miRNA sequence within the pri-miRNA is ~35kb. Many miRNAs appear to be very near to their promoters; for instance, the islet-enriched miRNAs miR-200c, miR-375, and miR-7, are ~0.5kb, ~1.5kb, and ~3.6kb from their respective promoters. Other miRNAs with important biological functions in the islet, such as miR-30e and miR-29b, are much further from their promoter (~45kb and ~65kb, respectively). Perhaps the most striking and unexpected finding was that miR-876/873 is ~325kb downstream of its promoter (Fig. **4**). This finding is supported by ESTs from human brain and pancreatic islet tis-sue.

The application of epigenomic strategies similar to the one described above in other human and mouse cell types has led to a comprehensive set of annotations for mammalian miRNA promoters [35], several of which have been validated by independent experiments [27, 28]. For example, Ozsolak et al. [27] performed ChIP-chip screens for H3K4me3, H3K9/14ac, RNA Polymerase II, and RNA Polymerase III in the genomic regions 20kb upstream and 1kb downstream of miRNAs in two melanoma cell lines and one breast cancer cell line. Their method accurately detected the few miRNA promoters that had been previously determined by 5’ rapid amplification of cDNA ends (RACE), including those for miR-146a, miR-146b, miR-155, and miR-21. They also additionally verified six novel miRNA promoters by promoter cloning and reporter gene assays. In another study, Barski et al. [28] performed ChIP-seq based profiling of eight histone methylation marks, including H3K4me3 and H3K79me2, to identify miRNA promoters in CD4+ T cells. They reported 85 miRNA promoters that are not shared with a protein-coding gene, among which >40% have ESTs that start at the predicted promoter and extend beyond the miRNA locus. They also additionally validated six novel miRNA promoters by 5’ RACE as well as by promoter cloning and reporter gene assays.

Most of the published epigenomic strategies for miRNA promoter identification are based on only three or four types of chromatin marks; however, there are over 100 known distinct histone modifications [36]. Recently, a computational biology group at the Massachusetts Institute of Technology used a multivariate Hidden Markov Model (HMM) to analyze previously published genome-wide profiles for 38 histone modifications in human T cells and defined 51 ‘chromatin states’ that correspond to specific functions, including several stages of promoter activity and transcription [37]. As similarly comprehensive epigenomic datasets become available for additional cell types, the HMM-based tool (ChromHMM) can be applied not only to identify high-confidence miRNA promoters, but also other types of regulatory elements (e.g. long-range enhancers or silencers) that likely contribute to miRNA transcription.

It is important to note that while the H3K4me3 mark is generally associated with promoters, it is not enriched at some non-constitutively active promoters [38]. To account for this, alternative strategies for promoter identification have emerged recently [39], including high-throughput sequencing of nascent RNA, which provides a snapshot of genome-wide transcriptional activity [40]. For example, an HMM was applied to nascent RNA-seq data from MCF-7 cells to determine the full-length primary transcripts of all expressed miRNAs and to assess which of these are regulated by estradiol [41]. 

## RELEVANT GENOMIC DATA REPOSITORIES

### microRNA Genomics

The official database of microRNAs, miRBase 18.0 [42], offers information on miRNAs in 168 different species. Currently, miRBase lists 1,921 mature human miRNAs. For each miRNA, miRBase provides the RNA sequence, genomic location (chromosomal coordinates), genomic context (overlapping genes), experimental evidence including data from high-throughput small RNA sequencing, and links to databases that list computationally predicted and/or empirically validated target genes. Despite the improvements brought about by high-throughput sequencing technology, accurate miRNA discovery remains technically challenging. As such, some sequences listed as candidate miRNAs in miRBase could represent other non-miRNA-related small RNA products (functional or not)[43]. Nonetheless, miRBase remains the most reliable, widely used and regularly updated database for miRNA-related information. Other related resources include miRGen, microRNA.org, miRNAMap, mimiRNA, TransmiR, miReg, and miRStart (see “Resource List”).

### The Epigenome

The NHGRI-funded Encyclopedia of DNA elements (ENCODE) Consortium and the NIH Roadmap Epigenomics Consortium manage repositories of genome-wide profiles of various types of biological data across a broad set of cell types and primary tissues.

At the present time, the Roadmap project has generated epigenomic data for 61 different cell/tissue types, with a particular focus on chromatin marks, including open chromatin, DNA methylation, and a variety of histone modifications. While not all of the different types of data are available for all of the cell types, epigenome-wide data for the following six histone modifications are available for all 61 cell types: H3K4me1, H3K4me3, H3K9me3, H3K27ac, H3K27me3, and H3K36me3. These histone modifications are referred to by the Roadmap project as the “core” set, and their relevance to chromatin structure and gene expression has been extensively discussed in several previous review articles. The Roadmap data can be visualized on and downloaded from numerous websites (see “Resource List”), including the Roadmap Browser, the Human Epigenome Atlas, the National Center for Biotechnology Information (NCBI) Epigenome Gateway, or the USCS Epigenome Browser. For further details on the Roadmap data resource, readers are directed to [44].

Currently, the ENCODE project has generated a diverse array of genomic information, including various epigenomic marks, transcription factor binding events, RNA-protein interactions, small RNA expression, mRNA expression, and three-dimensional chromatin structure, across ~150 different cell/tissue types. Although the scope of the ENCODE project is substantially broader than that of the Roadmap project, the number and types of data sets available across the different cell types is much more variable. For example, ENCODE has generated 335 different datasets for K562 cells (including 224 transcription factor and histone modification ChIP-seq datasets), but only 12, 4, and 2 datasets for human embryonic kidney 293 (HEK293) cells, primary hepatocytes, and human pulmonary artery endothelial cells (HPAEC), respectively. The data from the ENCODE Consortium is available for download directly from their website (http://en codeproject.org) and can be mined using the tools at the University of California at Santa Cruz (UCSC) Table Browser (see “Resource List”). For further details on the ENCODE project and related datasets, readers are directed to the Nature ENCODE Explorer (http://www.nature.com/encode) and the OpenHelix ENCODE Tutorial (http://www.openhelix.com/ENCODE).

Several tools, including RegulomeDB [45] and HaploReg [46], have been developed to map human genetic variants onto predicted regulatory elements as determined by integrative analysis of epigenomic data from the ENCODE and Roadmap projects [47]. These resources will be instrumental for characterizing functional elements that control miRNA transcription as well as for developing hypotheses regarding the molecular mechanisms underlying disease-associated genetic loci.

### Disease-Associated Genetic Variants

The NHGRI Genome Wide Association (GWA) Studies Catalog provides a centralized resource for Disease/trait-Associated Single nucleotide polymorphisms (DASs). As of June 2012, the catalog includes 1,271 GWA studies reporting 6,446 DASs, and the list is rapidly growing. Recent bioinformatic analyses of these data revealed that DASs are enriched in genomic regions that regulate gene expression [48, 49], particularly gene promoters [50]. Because changes in miRNA expression patterns have been previously correlated with a number of different phenotypes and disease outcomes [51], it is likely that DASs also occur in miRNA promoters. As more miRNA promoters are identified, they can be cross-referenced with DAS data from the GWAS Catalog in order to assess potential roles for transcriptional mis-regulation of miRNAs in complex disease etiology. For additional databases related to disease-associated genetic variants, see “Resource List”.

## CONCLUSION

miRNAs have emerged as important regulators of gene expression. While research in the area of miRNA-mediated gene regulation has blossomed in the last decade, the factors that regulate miRNA expression have remained elusive, in part due to the challenge of identifying regulatory elements that control miRNA transcription. However, the latest advances in genome technology and chromatin biology are enabling systematic functional annotation of the human genome, thereby shedding light on the previously obscured world of miRNA transcription.

Many large-scale efforts are currently underway to catalog epigenomic profiles of chromatin marks in a diverse array of cell types, in both normal and perturbed states. These databases represent rich resources for biological discovery. Integrative analysis of the data with tools such as ChromHMM will expand our knowledge base of functional regions of the genome, including miRNA promoters. As epigenomic data becomes available for a wider range of physiological conditions, it will be increasingly possible to investigate the plasticity of miRNA promoters.

Because a single miRNA can fine-tune the expression of hundreds to thousands of genes in numerous biological pathways, the mis-expression of miRNAs themselves likely underlies many inherited disorders. The recent GWAS finding that variants near miR-137 are implicated in schizophrenia is likely to be only the ‘tip of the iceberg’. As more miRNA promoters are identified, genetic variants that occur within them can be cross-referenced with a growing number of databases that provide information on disease-associated genetic loci.

## SIDEBAR 1: miRNA-RELATED GENETIC VARIANTS AND HUMAN DISEASE

miRNA-related cis-regulatory genetic variants (GVs) can occur within four categories: (1) mature miRNAs, (2) pre-miRNAs, (3) pri-miRNAs, or (4) miRNA transcriptional control elements (such as promoters). Population genomic analyses have demonstrated strong purifying selection on the first two functional categories, implying that GVs in mature miRNAs and pre-miRNAs are likely to be deleterious and could lead to disease [52, 53]. Several examples have been reported in the last few years, including a GV in pre-miR-16 that is associated with chronic lymphocytic leukemia [54] and two GVs, one in pre-miR-96 and the other in mature miR-96, that cause hearing loss [55, 56]. Until recently, the search for disease-associated GVs within categories 3 and 4 has been greatly impeded by a lack of well-defined annotations for these elements. One interesting case, which illustrates the potential impact of these categories of GVs, is that of a functional variant in the promoter of miR-146a [57] that confers significant risk for systemic lupus erythematosus [58].

A recent genome-wide association study of schizophrenia was the first large human population based study to report the significant association of a common single nucleotide polymorphism (SNP) at a miRNA locus (miR-137) with a complex disease [59]. Complex diseases such as schizophrenia are increasingly viewed as “network disorders”[60]. This makes it extremely challenging to identify disease-causing GVs in single genes, because biological networks encode mechanisms for conferring robustness against perturbations to individual genes within the network. Network robustness is imparted, in large part, by a web of miRNA activity [61]; as such, genetic perturbations of miRNAs will likely have a dramatic effect on network output. Therefore, it is expected that as more high-powered genetic association studies are performed, an increasing number of miRNA-related GVs will be implicated in complex disease etiology.

Several databases and web servers have been developed to facilitate data mining of GVs in miRNA-related genomic regions, including Patrocles, dbSMR, PolymiRTS, MicroSNiPer, miRdSNP, and dPORE-miRNA (see “Resource List”).

## RESOURCE LIST

Listed below are a few databases/web servers of relevance to this article’s discussion of microRNA genomics:

### microRNA Genomics

- miRBase: http://www.mirbase.org/
- miRGen: http://diana.cslab.ece.ntua.gr/mirgen/
- miRNAMap: http://mirnamap.mbc.nctu.edu.tw/
- microRNA.org: http://www.microrna.org/microrna/home.do
- mimiRNA: http://mimirna.centenary.org.au/mep/formulaire.html
- miReg: http://www.iioab-mireg.webs.com/
- TransmiR: http://202.38.126.151/hmdd/mirna/tf/
- miRStart: http://mirstart.mbc.nctu.edu.tw/

### Epigenomic Data

- ENCODE project: http://encodeproject.org
- UCSC Table Browser: http://genome.ucsc.edu/cgi-bin/hgTables
- Nature ENCODE Explorer: http://www.nature.com/encode
- UCSC Epigenome Browser: www.epigenomebrowser.org
- Roadmap Epigenomics Browser: www.roadmapepigenomics.org
- The Human Epigenome Atlas: http://www.genboree.org/epigenomeatlas
- NCBI Epigenome Gateway: http://www.ncbi.nlm.nih.gov/epigenomics
- RegulomeDB: http://regulome.stanford.edu/
- HaploReg: http://www.broadinstitute.org/mammals/haploreg/haploreg.php

### Disease-Associated Genetic Variants

- NHGRI GWAS Catalog: http://www.genome.gov/gwastudies/
- GWAS Integrator: http://hugenavigator.net/HuGENavigator/gWAHitStartPage.do
- GWASdb: http://jjwanglab.org:8080/gwasdb/
- OMIM: http://www.ncbi.nlm.nih.gov/omim

### miRNA-Related Genetic Variants

- Patrocles: http://www.patrocles.org/
- dbSMR: http://www.ncbi.nlm.nih.gov/pmc/articles/PMC 2676258/
- PolymiRTS: http://compbio.uthsc.edu/miRSNP/
- MicroSNiPer: http://cbdb.nimh.nih.gov/microsniper/
- miRdSNP: http://mirdsnp.ccr.buffalo.edu/
- dPORE-miRNA: http://cbrc.kaust.edu.sa/dpore/

## Figures and Tables

**Table 1.** Independent Promoter Regions for microRNAs in the Human Islet. Chromosomal Coordinates of Promoter Regions (TSS-500, TSS+1000) for ~50 pri-microRNAs in Independent Transcription Units Are Shown. The TSS is Defined As the Peak Point of the DNase I Hypersensitivity (DHS) Peak. For Every miRNA Cluster in the pri-miRNA, One Representative miRNA is Listed

microRNA	Promoter Location (hg18 build)
hsa-mir-99b	chr19:56882915-56886700
hsa-mir-193b	chr16:14302399-14305577
hsa-let-7a-1	chr9:95968156-95969786
hsa-mir-24-2	chr19:13810622-13815354
hsa-mir-606	chr10:76860686-76862025
hsa-mir-429	chr1:1087563-1092160
hsa-mir-339	chr7:1033001-1035203
hsa-mir-98	chrX:53726045-53729080
hsa-mir-125b-1	chr11:121473211-121477931
hsa-mir-200c	chr12:6941803-6944754
hsa-mir-365-2	chr17:26909754-26911504
hsa-mir-345	chr14:99820837-99822134
hsa-mir-1226	chr3:47841192-47842583
hsa-mir-640	chr19:19377081-19379270
hsa-mir-30b	chr8:135911379-135915052
hsa-mir-132	chr17:1898841-1901598
hsa-mir-221	chrX:45513235-45514697
hsa-mir-96	chr7:129251521-129253450
hsa-mir-101-1	chr1:65304088-65307746
hsa-mir-876	chr9:29200828-29206114
hsa-mir-130a	chr11:57161574-57163312
hsa-mir-210	chr11:557285-559766
hsa-mir-375	chr2:219573745-219576591
hsa-mir-129-1	chr7:127593751-127596151
hsa-mir-34a	chr1:9163794-9166232
hsa-mir-424	chrX:133510328-133512001
hsa-mir-760	chr1:94084075-94086554
hsa-mir-1470	chr19:15403286-15405395
hsa-mir-491	chr9:20673320-20675068
hsa-mir-148a	chr7:25955712-25959157
hsa-mir-27b	chr9:96850320-96852993
hsa-mir-187	chr18:31783792-31784578
hsa-mir-421	chrX:73426836-73430377
hsa-mir-137	chr1:98291509-98292780
hsa-mir-21	chr17:55269860-55274088
hsa-mir-29b-2	chr1:206107141-206109449
hsa-mir-505	chrX:138840991-138843422
hsa-mir-648	chr22:16862708-16864920
hsa-mir-1246	chr2:177210142-177211503
hsa-mir-181b-1	chr1:197171987-197173562
hsa-mir-1253	chr17:2598670-2600264
hsa-mir-1281	chr22:39746997-39749182
hsa-mir-181c	chr19:13844407-13845327
hsa-mir-30e	chr1:40946892-40948426
hsa-mir-1305	chr4:181215611-181218178
hsa-mir-551b	chr3:169448969-169451561
hsa-mir-1303	chr5:154042174-154043361
hsa-mir-149	chr2:241040110-241042229
hsa-mir-7-2	chr15:86948085-86950842

## ABBREVIATIONS

ChIP: = Chromatin immunoprecipitation
DAS: = Disease-Associated Single Nucleotide Polymorphism
DHS: = DNase I Hypersensitivity Site
ENCODE: = Encyclopedia of DNA Elements
GV: = Genetic Variant
GWAS: = Genome-Wide Association Study
H3K4me1: = Histone H3 lysine 4 Mono-methylation
H3K4me3: = Histone H3 lysine 4 tri-Methylation
H3K27ac: = Histone H3 lysine 27 Acetylation
H3K36me3: = Histone H3 lysine 36 tri-Methylation
H3K79me2: = Histone H3 lysine 79 di-methylation
H3K9me3: = Histone H3 lysine 9 tri-Methylation
H3K27me3: = Histone H3 lysine 27 tri-Methylation
HMM: = Hidden Markov Model
HPAEC: = Human Pulmonary Artery Endothelial Cells
K562: = Human Myelogenous Leukemia Cell Line
MCF-7: = Michigan Cancer Foundation-7 Breast Cancer Cell Line
miRNA: = microRNA
pre-miRNA: = miRNA Precursor
RACE: = Rapid Amplification of cDNA Ends
RISC: = RNA Induced Silencing Complex
SNP: = Single Nucleotide Polymorphism
TSS: = Transcription Start Site
